# Thirty years in, regulatory T cells are ready for medicine

**DOI:** 10.3389/fimmu.2026.1925870

**Published:** 2026-09-03

**Authors:** Ryoji Kawakami, Ayush Jain

**Affiliations:** 1 Experimental Pathology Lab, Institute for Life and Medical Sciences, Kyoto University, Kyoto, Japan; 2 Institute of Immunology, Universitätsklinikum Carl Gustav Carus, Technische Universität Dresden, Dresden, Germany

**Keywords:** autoimmune disease, cell therapy, Foxp3, immune tolerance, Treg - regulatory T cell

## Abstract

Regulatory T (Treg) cells are a specialized subset of CD4^+^ T cells indispensable for the establishment and maintenance of immunological tolerance. Treg cells employ diverse mechanisms of immune regulation mediated by a broad repertoire of immunosuppressive molecules under the control of the lineage-specifying transcription factor FoxP3. Functional defects in Treg cells disrupt immune homeostasis and are implicated in the pathogenesis of autoimmune diseases, chronic inflammatory disorders, and cancer. In this article, we revisit three decades of progress since Sakaguchi’s seminal discovery of CD25^+^ regulatory T cells in 1995, highlighting the physiological significance of dominant immune tolerance and its underlying molecular mechanisms. We then discuss how these mechanisms have inspired therapeutic strategies aimed at enhancing or harnessing Treg cell function, the remaining challenges and recent technological advances toward clinical translation, and the near-future prospects for Treg-based therapies in immune-mediated diseases.

## 1 Discovery of CD25^+^Foxp3^+^ regulatory T cells: a cellular and molecular basis for dominant tolerance

The immune system is a mechanism for eliminating harmful foreign antigens, such as those derived from pathogens, while establishing self-tolerance by avoiding responses to antigens that should be tolerated, including self-antigens. Since the mid-twentieth century, various hypotheses have been proposed to explain how T cells achieve such selective responsiveness. Broadly, these concepts can be divided into two categories. The first is cell-autonomous inactivation, represented by thymic negative selection and clonal anergy, both of which were experimentally demonstrated in the 1980s (1, 2). In this model, the responsiveness of antigen-reactive T cells is controlled within the responding T cells themselves: immature thymocytes are programmed to undergo apoptosis upon antigenic stimulation by self-antigens, or inappropriate antigen stimulation in the absence of co-stimulation results in impaired activation and proliferation. The second model is dominant tolerance, in which, among T cells reactive to a given antigen, a particular population suppresses cognate T cells with similar antigenic reactivity. Experimental studies based on sophisticated models of thymic selection and clonal anergy in the 1980s and early 1990s demonstrated the existence of these cell-autonomous mechanisms (3). These phenomena and the underlying experimental logic were fundamentally correct, and they are still recognized today as essential components of immune tolerance. However, at that time, whether the establishment and maintenance of self-tolerance depended solely on such mechanisms remained unresolved.

**Figure 1:**
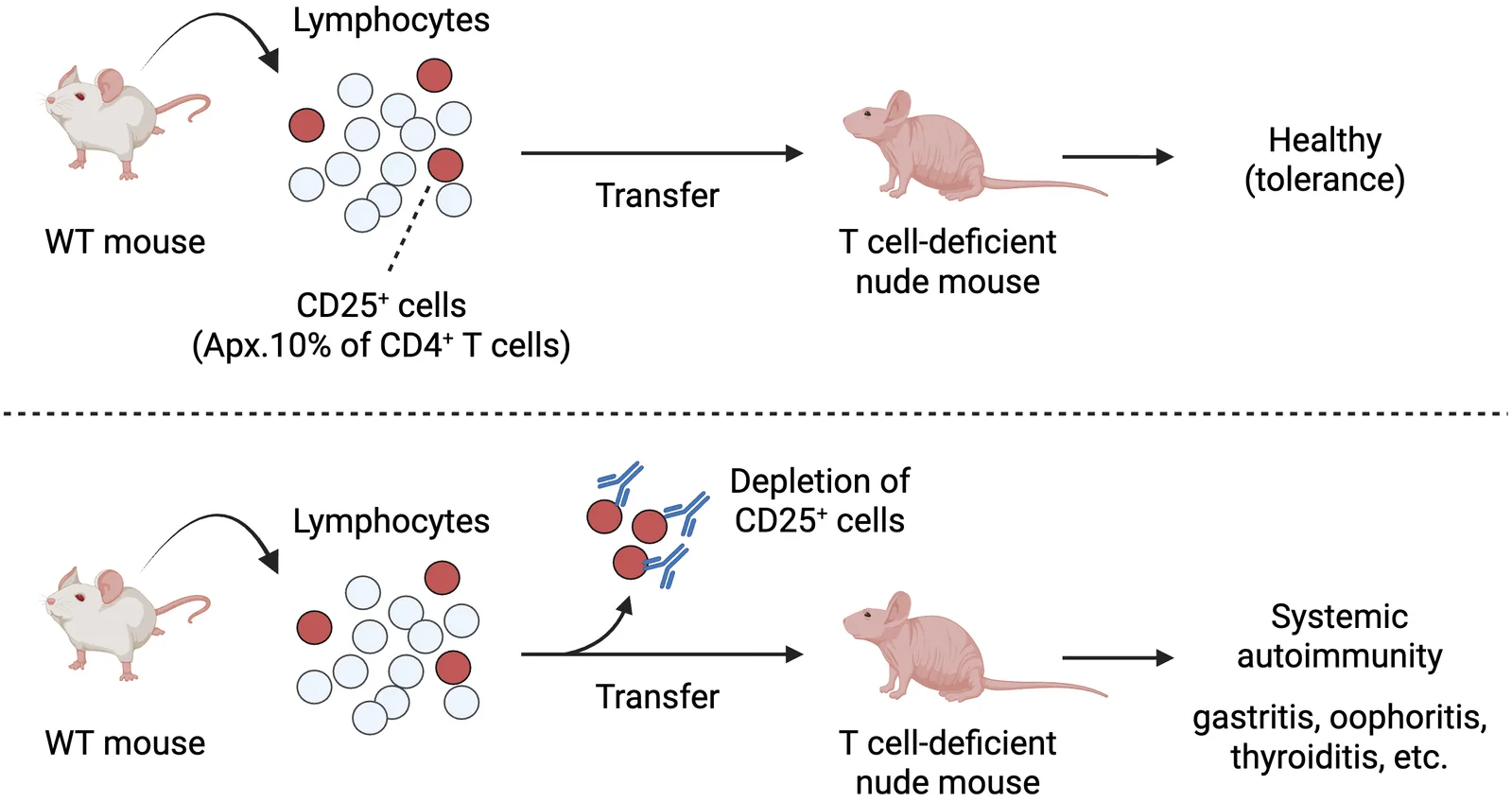
Demonstration of CD25^+^ regulatory T cells. Scheme of the experiment demonstrating the existence of CD25^+^ regulatory T cells, as shown in Sakaguchi et al., J. Immunol. 1995 (4). Transfer of lymphocytes from the spleen and lymph nodes of BALB/c mice into thymus-deficient nude mice does not cause immunological abnormalities. However, nude mice receiving lymphocytes after depletion of CD25^+^ cells develop a wide range of systemic autoimmune diseases, including gastritis, oophoritis, and thyroiditis. These findings demonstrated that CD25^+^ cells are essential for the maintenance of immunological tolerance.

In this context, Sakaguchi’s seminal 1995 report was pivotal in establishing that suppressive T cells exist physiologically and constitutively (4). Transferring mouse lymphocytes into T cell-deficient nude mice normally does not cause immunological abnormalities. However, the study showed that when a small population of CD4^+^ T cells expressing CD25, accounting for only approximately 10% of CD4^+^ T cells, was removed, the recipient T cell-deficient mice developed systemic autoimmune diseases. This clearly demonstrated that CD25^+^ cells play a critical role in the maintenance of immune tolerance (**Figure 1**). Subsequent confirmatory studies, including those by the Sakaguchi and E. M. Shevach groups, clarified the major characteristics of CD25^+^CD4^+^ T cells. These cells were found to highly express the high-affinity IL-2 receptor and the immunosuppressive molecule CTLA-4, and to be produced in substantial numbers in the thymus in a functionally mature state (5–7). Although CD25 expression can be readily induced *in vitro* by TCR stimulation, such induced CD25^+^ cells do not possess suppressive activity, suggesting that CD25^+^CD4^+^ T cells present under physiological conditions represent a distinct cell lineage (8, 9). By around 2000, this population had come to be referred to as “regulatory” T cells (10).

The identification of FoxP3 as the master transcription factor of regulatory T (Treg) cells in 2003 provided definitive evidence that a distinct lineage of CD4^+^ T cells specialized for immune suppression is constitutively embedded within the acquired immune system. The FOXP3 gene was identified as the causative gene for the Scurfy mouse phenotype and for human IPEX syndrome, which is characterized by spontaneous systemic autoimmune inflammation early in life (11, 12). FoxP3 is predominantly expressed in CD25^+^CD4^+^ T cells and functions as the master transcription factor of Treg cells. Introduction of FOXP3 into conventional CD4^+^ T cells induced the expression of Treg-signature genes such as CD25 and CTLA-4 and conferred immunosuppressive activity (13–15). Loss-of-functional mutations in FOXP3 or experimental depletion of Foxp3^+^ Treg cells at any point in life were shown to induce fatal systemic autoimmune disease, demonstrating that Treg-mediated immune tolerance is constitutively required (16–19). These findings demonstrated that even under physiological conditions, the immune system constitutively harbors self-reactive T cells that cannot be fully controlled by thymic selection and clonal anergy, and that dominant tolerance mediated by Treg cells is indispensable for the establishment and maintenance of immune tolerance. The identification of CD25 and FoxP3 as shared molecular markers between humans and mice facilitated analyses of Treg cells in clinical disease settings. Accumulating studies showed that Treg cell number and function are associated with the pathogenesis of cancer and autoimmune diseases in humans, and that adoptive transfer of Treg cells can ameliorate disease phenotypes in animal models of autoimmunity and transplantation (20–22). Building on these findings, the prospect of leveraging Treg cells and their regulatory functions to restore dominant tolerance in immune-mediated diseases has gained considerable momentum, spurring a new wave of mechanistic studies alongside parallel efforts to bring these insights into the clinic.

## 2 Cardinal biological features of Treg cells

**Figure 2:**
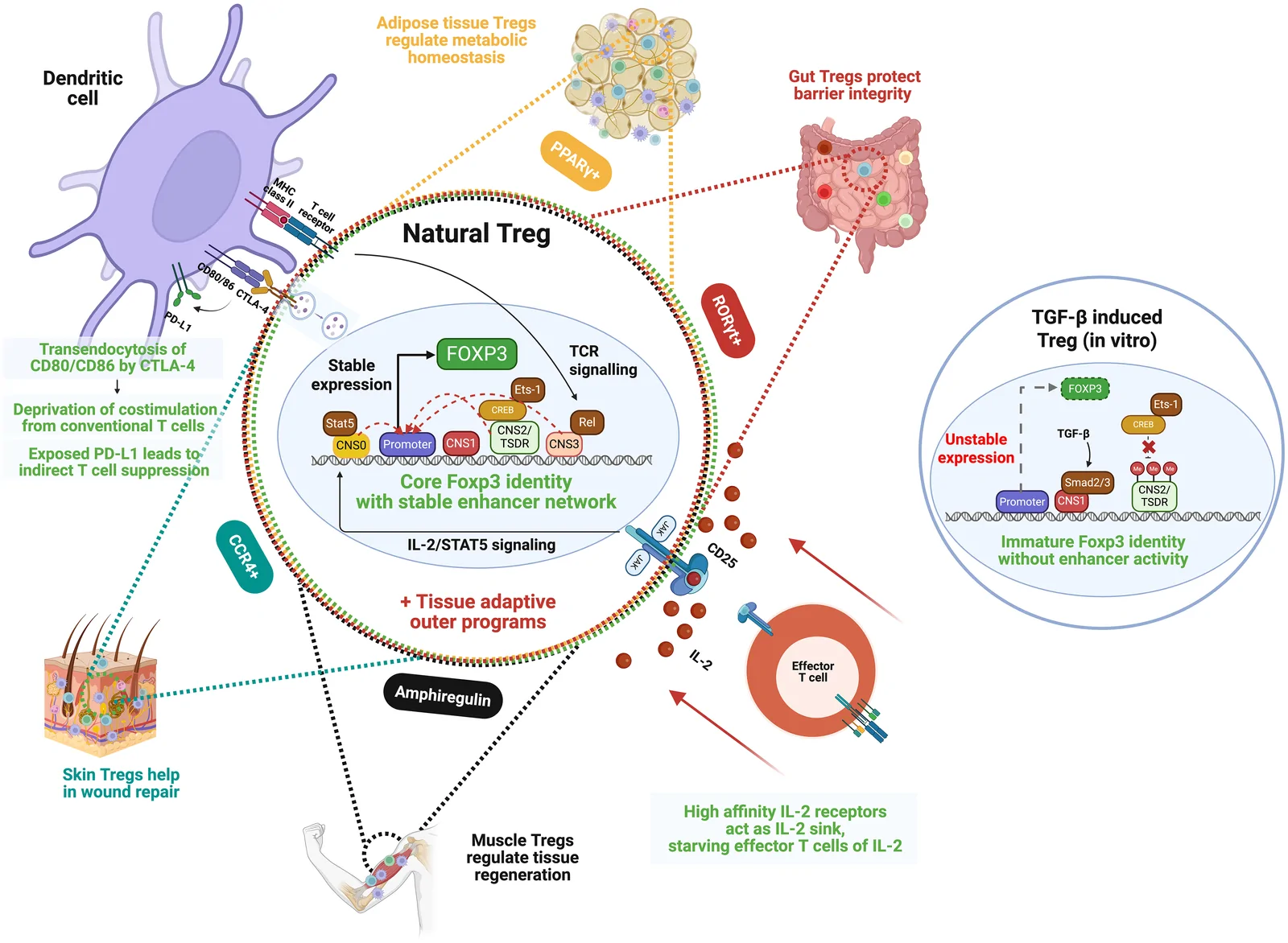
Three interdependent pillars of regulatory T cell biology. Schematic representation of the three biological pillars that collectively define Treg identity and function, organized around a central Treg cell. The Treg is depicted with a dual-layered border: the inner solid circle represents the core Foxp3-dependent identity shared by all Tregs, while the outer multicolored dashed circle represents the tissue-adaptive transcriptional programs acquired upon residency in non-lymphoid tissues, layered on top of, not substituted for, the core regulatory identity. On the left, Treg-expressed CTLA-4 captures and removes CD80/CD86 from dendritic cells via transendocytosis, simultaneously exposing PD-L1 and enabling indirect suppression through the PD-1 axis. On the right, the high-affinity IL-2 receptor (CD25/CD122/CD132) allows Tregs to act as an IL-2 sink, depriving effector T cells of this essential growth factor. Within the nucleus, the *Foxp3* locus is shown in two epigenetic states: natural Tregs (left), the distal enhancer CNS0 integrates IL-2/STAT5 signaling and engages in genomic looping with CNS3 and the promoter to initiate Foxp3 transcription; the Treg-specific demethylated region (TSDR/CNS2) is fully demethylated, permitting transcription factor binding (CREB, Ets-1) and stable Foxp3 expression; in TGF-β-induced Tregs in vitro (right), the TSDR remains methylated, blocking transcription factor access and resulting in unstable Foxp3 expression. This enhancer looping visually connects the IL-2 consumed at the cell surface to the epigenomic machinery controlling Treg identity in the nucleus. The outer border connects to non-lymphoid tissue compartments in which Tregs acquire specialized identities: visceral adipose tissue (PPARγ+, metabolic homeostasis), gut (RORγt+, barrier integrity), skin (CCR4+, wound repair), and muscle (amphiregulin+, tissue regeneration).

The identification of Foxp3 as the lineage-defining transcription factor gave the field, for the first time, a molecular entry point into Treg biology (13–15, 19). But knowing what marks a Treg raised a deeper set of questions, such as how do Tregs actually suppress? What keeps them committed to their suppressive identity over a lifetime? And how do they adapt to the vastly different immunological demands of distinct tissues? The work that followed over the next two decades addressed each of these questions and, in doing so, uncovered the most fundamental principles of Treg biology (**Figure 2**).

The fundamental insights into the suppressive activity came with the recognition of CTLA-4 as a negative regulator of T cell activation, well before its connection to Tregs became clear. The decisive experiment came from Wing and colleagues in the Sakaguchi laboratory, who generated mice with a Treg-specific CTLA-4 deletion. These animals developed fatal systemic autoimmunity within weeks, experiencing lymphoproliferation, tissue destruction, and massive IgE production, demonstrating that CTLA-4 is non-redundant for Treg suppressive function *in vivo* (23). Critically, the study showed that Treg-expressed CTLA-4 directly downregulates the costimulatory ligands CD80 and CD86 on dendritic cells, effectively disarming them before they can activate naïve T cells. How CTLA-4 achieves this became clearer when Qureshi and Sansom described transendocytosis, the physical capture and internalization of CD80/CD86 from the surface of antigen-presenting cells (24). This was not merely competitive binding but active ligand removal. More recently, Tekguc, Wing, and Sakaguchi extended this model by showing that stripping CD80/CD86 simultaneously liberates free PD-L1 on dendritic cells, connecting CTLA-4-mediated suppression to the PD-1 checkpoint pathway (25). This biology has direct therapeutic relevance in both directions: anti-CTLA-4 antibodies like ipilimumab unleash antitumor immunity in part by disabling Treg suppression, while the CTLA-4-Ig fusion protein abatacept mimics what Tregs do naturally, by blocking co-stimulation to dampen autoimmune responses (26).

While CTLA-4 defines how Tregs exert suppression, their ability to persist depends critically on IL-2. Paradoxically, IL-2, originally characterized as a growth factor for effector T cells, turned out to be most critical for the cells that restrain them. Tregs constitutively express the high-affinity trimeric IL-2 receptor (CD25, CD122, CD132), but are intrinsically wired not to produce any IL-2 themselves, making them entirely dependent on paracrine supply for survival, proliferation, and functional competence. Fontenot and colleagues from the Rudensky laboratory showed that disrupting this supply leads to Treg collapse and spontaneous autoimmunity (27). This dependence has been exploited therapeutically through low-dose IL-2 administration, which preferentially expands Tregs over effector T cells owing to their higher receptor affinity. Two back-to-back clinical studies in 2011 validated this idea. Koreth et al. showed that low-dose IL-2 expanded functional Tregs and improved outcomes in chronic graft-versus-host disease (28), while Saadoun et al. demonstrated similar results in hepatitis C-associated vasculitis (29). However, the practical limitations of native IL-2 soon became apparent, primarily due to its short serum half-life, which requires frequent dosing, and some activation of NK and effector T cells occurs even at low doses. These constraints have driven the development of next-generation IL-2 muteins engineered for enhanced Treg selectivity, such as the IgG-(IL-2N88D)_2_ fusion protein, which achieves sustained Treg expansion in primates without detectable effector T cell activation (30), and mRNA-encoded IL-2 muteins with comparable selectivity (31).

Interestingly, neither CTLA-4-mediated suppression nor IL-2-driven survival matters much if a Treg loses its identity and converts into a pro-inflammatory effector cell. The question of what stabilizes Treg commitment led to one of the most important conceptual shifts in the field, the recognition that Foxp3 expression alone is insufficient for durable Treg identity. The key insight came from Huehn and colleagues, who identified an evolutionarily conserved CpG-rich element within the *Foxp3* locus, which was later termed the Treg-specific demethylated region (TSDR), that is fully demethylated in thymus-derived Tregs but remains methylated in naïve T cells and, critically, only partially demethylated in TGF-β-induced Foxp3^+^ T cells (32). This epigenetic asymmetry explained the long-standing puzzle: why *in vitro*-generated Tregs, despite robust Foxp3 expression, lose suppressive function upon restimulation, while thymus-derived Tregs maintain stable identity. The answer was not in the transcription factor itself, but in the epigenetic state of its locus. Subsequent work from the same group showed that specific CpG motifs within the TSDR gate access to transcription factors, including CREB/ATF and Ets-1, providing a molecular mechanism for how demethylation locks in Foxp3 transcription (33, 34). Complementing this work on the *Foxp3* locus, Rudensky and colleagues dissected the functional roles of three conserved non-coding elements (CNS1-3), showing that CNS2, which overlaps with the TSDR, is specifically required for heritable Foxp3 expression during cell division. At the same time, CNS3 acts as a pioneer element for initial Foxp3 induction in the thymus (35). Notably, Williams and Rudensky had shown earlier that even in cells with established epigenetic marks, conditional Foxp3 ablation leads to loss of suppressive function and acquisition of effector properties (36), indicating that epigenetic programming and sustained Foxp3 expression work as complementary safeguards, not redundant ones. The regulatory landscape of the *Foxp3* locus was further expanded with the identification of CNS0, a distal enhancer located upstream of the promoter (37, 38). Three independent studies converged on this element from different angles. Dikiy et al. showed that CNS0 serves as the molecular node through which IL-2-STAT5 signaling directly drives Foxp3 induction, and that its deficiency impairs neonatal Treg generation and exacerbates autoimmunity in the context of Aire deficiency (39). In a companion study, Kawakami et al. demonstrated that CNS0 and CNS3 are sequentially activated during early thymocyte differentiation, engaging in genomic looping with the Foxp3 promoter, and combined deletion of both elements completely abrogated Treg generation and caused fatal systemic autoimmunity (40). Zong et al. further showed that CNS0 and CNS3 synergize during Foxp3 induction, with CNS0 integrating IL-2/STAT5 signaling and CNS3 integrating TCR signals, while CNS0 and CNS2 cooperate to maintain Foxp3 expression during homeostatic expansion (41). Collectively, these findings revealed that CNS0 functions as the critical upstream enhancer that links IL-2 receptor signaling to the epigenomic machinery controlling Foxp3 transcription, thereby connecting two of the three biological pillars discussed here. Most recently, Umhoefer et al. extended these findings to the human *FOXP3* locus using large-scale CRISPRi tiling screens, confirming that CNS0 is essential for maintaining FOXP3 expression in human Tregs and identifying additional cell-type-specific regulatory elements that distinguish Treg enhancer usage from that of conventional T cells (42).

A pivotal study from the Sakaguchi laboratory brought these threads together by demonstrating that TCR-driven epigenetic remodeling at Treg signature gene loci and Foxp3 induction are independent, complementary events during thymic Treg differentiation (43). This insight led to a conceptual reframing from a “Foxp3-centered” to an “epigenome-defined” view of Treg identity (44). The clinical weight of this concept became apparent when Ohkura and colleagues characterized genome-wide epigenomic profiles of human Tregs and found that autoimmune disease-associated SNPs are concentrated and enriched roughly ninefold in Treg-specific super-enhancers at loci including *FOXP3*, *CD25*, and *CTLA4* (45). This finding provided a direct mechanistic link between Treg epigenomic integrity and genetic susceptibility to common autoimmune disease and thus carries important implications for therapeutic strategies that aim to generate or expand Tregs.

Beyond the molecular regulation of Treg identity, the past decade has revealed that Tregs residing in non-lymphoid tissues are not simply circulating Tregs that have settled in a new location, but have acquired specialized identities that go well beyond classical suppression. The prime example was the discovery of visceral adipose tissue Tregs expressing PPAR-γ by Feuerer, Mathis, and Benoist (46, 47). These Tregs regulate metabolic homeostasis in ways that extend well beyond immunosuppression and establish that Tregs acquire tissue-adapted identities with specialized functions. Tissue Tregs have since been described across skin, muscle, lung, and gut, each with distinct transcriptional programs layered on the core Foxp3-dependent identity (48, 49). A comprehensive review by Sakaguchi and colleagues further integrated the tissue Treg concept within the broader framework of Treg-mediated disease control, emphasizing that the phenotypic and functional heterogeneity of Tregs across anatomical compartments has direct implications for understanding how Treg dysfunction contributes to organ-specific autoimmune pathology (20). For therapeutic development, this heterogeneity means that Treg-based treatments may need to be tailored to the tissue context in which suppression, or repair, is required.

Overall, the biology of Tregs rests on three interdependent pillars: effector mechanisms centered on CTLA-4 and IL-2, epigenomic programming that ensures lineage fidelity, and tissue-specific differentiation that matches regulatory function to local demand. Each of these pillars now offers a distinct point of therapeutic intervention, a translation from basic biology to medicine that we consider next.

## 3 Targeting Treg biology for therapy in autoimmune diseases and transplantation

Since the discovery of Treg cells, three decades of investigation have transformed our understanding of immune tolerance and disease. Elucidation of the molecular mechanisms governing Treg-cell development, stability, and suppressive function has not only revealed their central role in maintaining immune homeostasis but has also provided a conceptual framework for therapeutic intervention. Treg cells are uniquely equipped to recognize antigen and proliferate while concurrently suppressing neighboring immune responses through multiple mechanisms, including deprivation of co-stimulatory signals and dominant consumption of IL-2. Furthermore, they adapt to diverse tissue and inflammatory environments while stably preserving lineage identity and suppressive capacity over long periods. Therefore, Treg cells have been an attractive therapeutic target and platform, leading to the development of strategies that either augment endogenous Treg activity or employ ex vivo-manipulated Treg products, several of which have now entered clinical evaluation and application.

Modulation of the Tconv/Treg balance through selective activation, functional enhancement, or direct supplementation of Treg cells by adoptive transfer has been expected as a promising approach for controlling autoimmune diseases and induction of transplantation tolerance. Since Treg cells primarily consume IL-2 in the environment by their constitutive expression of high-affinity IL-2 receptor alpha subunit CD25, low-dose IL-2 therapy has been investigated, aiming at increasing the Treg/Tconv ratio in settings such as T1D, Graft-versus-Host disease (GvHD), and atopic dermatitis (28, 50–52). Mice models and several preclinical trials have yielded several promising results; however, the challenge remains that it is difficult to determine an appropriate IL-2 dosage that can selectively boost Treg cells without activating Tconv cells. A more direct approach is Treg cell therapy, in which Treg cells themselves are administered to patients. Pioneering trials using ex vivo-expanded autologous Treg transfer were conducted in pediatric T1D patients, demonstrating safety and showing that lower ex vivo fold expansion of the Treg product was associated with preserved C-peptide production following treatment (53, 54). Although these studies represent important milestones in the clinical translation of Treg-based therapies, they have not yet demonstrated dramatic therapeutic efficacy (55). Nevertheless, continuous advances in our understanding of Treg biology and the rapid development of synthetic biology hold huge potential to establish Treg-based therapies as more fundamental and powerful treatments for immune-mediated diseases.

To overcome the problem of nonspecific T-cell activation associated with IL-2 therapy, substantial efforts have been devoted to the development of IL-2 muteins that preferentially stimulate Treg cells. IL-2 variants with enhanced selectivity for CD25 (IL-2Rα), highly expressed on Treg cells, relative to IL-2 receptor β/γ chains have been identified, and their enhanced therapeutic efficacy has been suggested in mouse models such as T1D (56, 57). Another important point is that Treg-mediated suppression generally requires antigen recognition, posing a possibility that mismatches between the antigen specificity of infused Treg cells and that of pathogenic T cells may restrict therapeutic efficacy. One potential solution is the application of chimeric antigen receptor (CAR) technology, which is based on designer synthetic molecules composed of antibody-derived recognition domains linked to T-cell signaling modules. By introducing CARs into Treg cells, these engineered cells can be selectively activated upon encountering target antigens and exert potent immunosuppressive functions (58). For example, CD19-CAR Treg cells and HLA-A2-CAR Treg cells have been reported to ameliorate disease pathology in mouse models such as GvHD, SLE, and transplantation (59–63). In these mouse models, CAR-Treg cells exerted their therapeutic effects through immunosuppressive activity rather than cytotoxicity, suggesting a potentially safer and mechanistically distinct alternative to conventional CD19-CAR-T-mediated B-cell depletion (60, 62). In studies applying HLA-A2-CAR Treg cells to islet transplantation in NOD mice, not only were alloimmune responses against the graft suppressed, but induction of Treg cells specific for endogenous islet-reactive T cells was also observed (63). These findings suggest that antigen-specific Treg therapy may promote linked suppression extending beyond the primary target antigen, thereby potentially broadening antigen-specific tolerance.

However, the causative antigens and the pathogenic TCRs are largely unknown in many autoimmune diseases, posing a challenge for stratifying “antigen-specific” therapeutics using such technologies of synthetic biology. To overcome this limitation, another possible strategy is the direct conversion of pathogenic effector/memory T cells in the patients into Treg cells. Given that patient-derived effector/memory T cells enrich the pathogenic T cell clone, their conversion into Tregs could be a feasible approach to treat autoimmune diseases even without knowledge of disease-causative antigens (64). Until recently, it has been challenging to generate nTreg-like stable Treg cells with a Treg-type epigenome with conventional *in vitro* Treg induction methods that use agonistic TCR stimulation, Co-stimulation, IL-2, and TGF-β (33, 43). However, identification of ascorbate as a critical activator of DNA demethylation enzyme TET2/3 for establishment of Treg-epigenome (65, 66), together with the optimization of quantitative and temporal regulation of TCR/co-stimulation/IL-2 signaling, has enabled conversion of patients-derived pathogenic effector/memory T cells into stable, nTreg-like cells with epigenetic identity and suppressive function (67, 68). Indeed, the therapeutic efficacy of antigen-specific induced Treg cells has recently been demonstrated in a mouse model of pemphigus vulgaris (69), providing compelling proof-of-concept for the clinical development of antigen-specific Treg cell therapies.

Importantly, IL-2 therapy, natural Treg-cell transfer, CAR-Treg technology, and Treg conversion therapies are not mutually exclusive approaches. Rather, their rational combination may synergistically promote both the induction and long-term maintenance of antigen-specific immune tolerance. One notable example is the orthogonal IL-2/IL-2 receptor system, in which engineered IL-2 and IL-2 receptor pairs selectively interact with each other while remaining insulated from endogenous IL-2 signaling (70). By engineering Treg cells to express an orthogonal IL-2 receptor and administering the cognate orthogonal IL-2, transferred Treg cells can be selectively expanded and activated *in vivo* (71, 72). More recently, engineered Treg cells have been generated that secrete their own orthogonal IL-2, creating an autonomous cytokine circuit that supports their selective persistence and expansion (73, 74). Such combinatorial approaches are likely to further accelerate the development of Treg-based therapeutics grounded in the fundamental principles of Treg biology.

To translate these promising findings from preclinical animal models into human Treg cell therapies, careful safety evaluation will be essential. To date, no serious adverse events attributed to Treg cell infusion have been reported in clinical trials of Treg cell therapy. However, previous studies have shown that a substantial proportion of the transferred Treg cells were lost shortly after administration (53, 75, 76). Therefore, if the technological advances discussed in this article enable the prolonged persistence of transferred Treg cells *in vivo*, their long-term safety will need to be carefully evaluated, with respect to their phenotypic and functional stability in inflammatory environments and the risk of non-specific immunosuppression. In addition, rigorous pharmaceutical quality control will also be essential to ensure safety. Reliable manufacturing process must be established to guarantee the purity of Treg cell products and, in approaches involving Treg conversion, the completeness and reproducibility of the conversion process.

## 4 Future prospects of Treg-mediated immunological therapy

The ultimate therapeutic goal in autoimmune diseases and transplantation is not merely transient disease control, but the stable establishment and maintenance of antigen-specific immune tolerance. To develop Treg-based therapies into robust and durable approaches for the induction of long-lasting immune tolerance, it will be essential to understand not only the intrinsic biology of Treg cells but also the mechanisms underlying the establishment and maintenance of tolerogenic niches and tolerance-inducing cellular ecosystems that support their generation, function, and persistence. Recent studies have identified specialized antigen-presenting cell populations in the intestine that selectively induce Treg cells in response to commensal microorganisms and dietary antigens (77–80). Over the next decade, it will be important to comprehensively elucidate such environmental factors that support Treg-mediated tolerance and to translate such findings into therapeutic strategies. Recent immunomodulatory antigen-delivery technologies may offer an approach for controlling such tolerogenic environments; e.g., noninflammatory lipid nanoparticle-formulated mRNA encoding myelin autoantigens has been shown to induce antigen-specific Treg cells and establish immune tolerance in an experimental model of multiple sclerosis (81). Thirty years after the discovery of Treg cells, the field is moving beyond the manipulation of Treg cells toward the engineering of tolerance-supportive immune ecosystems. Such advances may ultimately enable precise therapeutic control of antigen-specific dominant tolerance across a broad spectrum of immune-mediated diseases.
